# Biofilm Formation Drives Transfer of the Conjugative Element ICE*Bs1* in *Bacillus subtilis*

**DOI:** 10.1128/mSphere.00473-18

**Published:** 2018-09-26

**Authors:** Frédéric Lécuyer, Jean-Sébastien Bourassa, Martin Gélinas, Vincent Charron-Lamoureux, Vincent Burrus, Pascale B. Beauregard

**Affiliations:** a Département de Biologie, Faculté des Sciences, Université de Sherbrooke, Sherbrooke, Quebec, Canada; University of Iowa

**Keywords:** *Bacillus subtilis*, ICE*Bs1*, biofilms, extracellular matrix, horizontal gene transfer

## Abstract

Horizontal gene transfer by integrative and conjugative elements (ICEs) is a very important mechanism for spreading antibiotic resistance in various bacterial species. In environmental and clinical settings, most bacteria form biofilms as a way to protect themselves against extracellular stress. However, much remains to be known about ICE transfer in biofilms. Using ICE*Bs1* from Bacillus subtilis, we show that the natural conjugation efficiency of this ICE is greatly affected by the ability of the donor and recipient to form a biofilm. ICE*Bs1* transfer considerably increases in biofilm, even at low donor/recipient ratios. Also, while there is a clear temporal correlation between biofilm formation and ICE*Bs1* transfer, biofilms do not alter the level of ICE*Bs1* excision in donor cells. Conjugative transfer appears to be favored by the biophysical context of biofilms. Indeed, extracellular matrix production, particularly from the recipient cells, is essential for biofilms to promote ICE*Bs1* transfer. Our study provides basic new knowledge on the high rate of conjugative transfer of ICEs in biofilms, a widely preponderant bacterial lifestyle in the environment, which could have a major impact on our understanding of horizontal gene transfer in natural and clinical environments.

**IMPORTANCE** Transfer of mobile genetic elements from one bacterium to another is the principal cause of the spread of antibiotic resistance. However, the dissemination of these elements in environmental contexts is poorly understood. In clinical and environmental settings, bacteria are often found living in multicellular communities encased in a matrix, a structure known as a biofilm. In this study, we examined how forming a biofilm influences the transmission of an integrative and conjugative element (ICE). Using the model Gram-positive bacterium B. subtilis, we observed that biofilm formation highly favors ICE transfer. This increase in conjugative transfer is due to the production of extracellular matrix, which creates an ideal biophysical context. Our study provides important insights into the role of the biofilm structure in driving conjugative transfer, which is of major importance since biofilm is a widely preponderant bacterial lifestyle for clinically relevant bacterial strains.

## Abstract (precis)

Transfer of mobile genetic elements from one bacterium to another is the principal cause of the spread of antibiotic resistance. However, the dissemination of these elements in environmental contexts is poorly understood. In clinical and environmental settings, bacteria are often found living in multicellular communities encased in a matrix, a structure known as a biofilm. In this study, we examined how forming a biofilm influences the transmission of an integrative and conjugative element (ICE). Using the model Gram-positive bacterium B. subtilis, we observed that biofilm formation highly favors ICE transfer. This increase in conjugative transfer is due to the production of extracellular matrix, which creates an ideal biophysical context. Our study provides important insights into the role of the biofilm structure in driving conjugative transfer, which is of major importance since biofilm is a widely preponderant bacterial lifestyle for clinically relevant bacterial strains.

## INTRODUCTION

Acquisition of genetic material via horizontal gene transfer (HGT) is a fundamental phenomenon for bacterial adaptation and evolution (1). Conjugation, which is regarded as the broadest and most efficient mechanism of HGT, allows bacteria to transfer genetic material such as conjugative plasmids and integrative and conjugative elements (ICEs) through direct cellular contact (2). These mobile genetic elements are autonomous since they encode their own mating apparatus. They often contain genes responsible for a wide range of functions, including virulence, antibiotic resistance, and symbiosis (3–5). Conjugative plasmids and ICEs can often transfer between different bacterial species and genera and mobilize genomic islands or plasmids that are otherwise not self-transmissible, granting these elements an extensive role in bacterial evolution (6–9).

ICE*Bs1* is a 20.5-kb ICE that is present in many strains of Bacillus subtilis (10, 11), a low-G+C Gram-positive bacterium that is well studied for its plant growth-promoting effect (12–14). While ICE*Bs1* can mobilize genetic elements lacking mobilization functions (8), whether it provides any advantage for its host cell remains unclear (15). ICE*Bs1* transmission is initiated in the donor cell by its excision from the 3′ end of the chromosomal *trnS-leu2* gene (10). The resulting double-stranded circular intermediate undergoes rolling circle replication initiated at the origin of transfer (*oriT*) by the relaxase NicK, which cleaves the DNA strand to be transferred (16, 17). The nicked strand of ICE*Bs1* is then translocated into the recipient cells by an ICE*Bs1*-encoded type IV secretion system (17, 18). In the recipient cell, the transferred strand is recircularized, and its complementary strand is synthesized. The circular copy of ICE*Bs1* then integrates at the 3′ end of *trnS-leu2*, the *attB* site of the chromosome of the recipient (19).

Interestingly, two distinct cellular pathways regulate ICE*Bs1* excision. One is the global DNA damage response, which is mediated via the DNA repair protein RecA, which acts as an activator of conjugation. The other pathway is the ICE*Bs1*-encoded quorum-sensing system RapI-PhrI, consisting of RapI, an inducer of ICE*Bs1* excision that can be inhibited by the coexpressed oligopeptide PhrI (10), which is secreted in the extracellular environment and imported back into the cell through a permease. In this pathway, ICE*Bs1* excision is repressed in a community where ICE*Bs1*-harboring cells are widely present since the extracellular PhrI level is sufficient to inhibit RapI (10).

Biofilms are microbial communities surrounded by an extracellular matrix that protects bacterial cells from external stressors such as antibiotics and heavy metals (20, 21). In the environment and during chronic infections, most bacteria live within biofilms (22). B. subtilis biofilm matrix is mostly composed of exopolysaccharides and amyloid-like fibers, synthesis of which is encoded by the *epsA* to -*O* (*epsA–O*) operon and the *tapA-sipW-tasA* operon, respectively (23). Matrix production, and thus, biofilm formation, is triggered by a variety of environmental and physiological signals, including the lipopeptide surfactin, plant polysaccharides, and a combination of glycerol and manganese (24–26). In a planktonic population, the expression of the matrix production operons *epsA–O* and *tapA-sipW-tasA* is inhibited by the transcriptional repressor SinR (27).

Studies have suggested that biofilms are hot spots for the transfer of conjugative plasmids due to the high proximity of cells within this multicellular structure, but the importance of the extracellular matrix in this process is unexplored (28, 29). Also, many of the pathogens that have acquired antibiotic resistance through conjugative elements can form biofilms (30–32). However, despite their fundamental importance in antibiotic resistance gene dissemination, ICE propagation in biofilms has not yet been examined. Here, we take advantage of the extensive knowledge of ICE*Bs1* and B. subtilis biofilms to evaluate the dynamics of ICE*Bs1* dissemination within biofilms. Using medium that does or does not induce biofilm formation as support for conjugation, we report here that natural ICE*Bs1* transmission is 100- to 10,000-fold more efficient when cells are forming a biofilm, even when recipient cells outnumber donor cells. However, while biofilm formation increases conjugation, it does not influence ICE*Bs1* excision, suggesting that its effect occurs at the contact level. Accordingly, we observed that the biofilm extracellular matrix is crucial for enhanced ICE*Bs1* transfer in biofilms.

## RESULTS

### Biofilm enhances the conjugative transfer of ICE*Bs1*.

**FIG 1:**
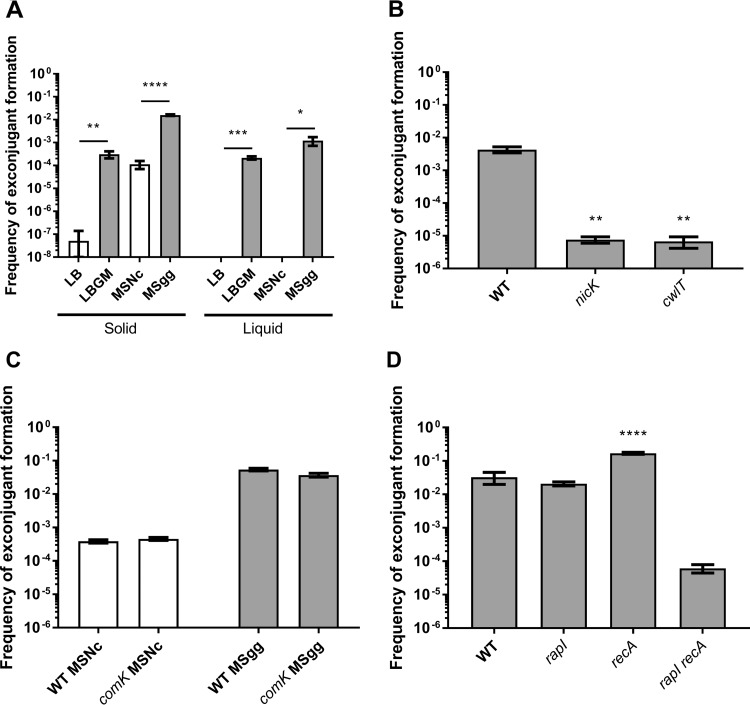
Biofilm formation enhances ICE*Bs1* transfer. (A) Donor cells with a kanamycin resistance cassette inserted in ICE*Bs1* were mated with recipient cells bearing an intergenic chloramphenicol resistance cassette in a 1:1 ratio on non-biofilm-inducing solid and liquid media (LB and MSNc [white bars]) and biofilm-inducing solid and liquid (pellicle-inducing) media (LBGM and MSgg [gray bars]). Statistical analysis indicates a significant increase in ICE*Bs1* transfer when biofilm is formed (Student’s *t* test; *, *P* < 0.05; **, *P* < 0.01; ***, *P* < 0.001; ****, *P* < 0.0001). (B) Conjugation-deficient donor cells (*nicK* and *cwlT*) were mated with WT cells on MSgg to assess mating efficiency. Statistical analysis shows a significant decrease in mating efficiency with the *nicK* and *cwlT* donor cells (one-way ANOVA; **, *P* < 0.01). (C) Transformation-deficient cells (*comK*) were mated on MSNc and MSgg, and mating efficiency was compared to that of WT cells. Statistical analysis shows no significant difference in mating efficiencies between *comK* and WT cells (Student’s *t* test). (D) Single and double mutant ICE*Bs1* activation pathway donor cells were mated with WT recipient cells on MSgg. Statistical analysis shows a significant increase of ICE*Bs1* mating efficiency between *recA* and WT donor cells, but not with the *rapI* mutant (one-way ANOVA; ****, *P* < 0.0001). While the double mutant showed a decrease in mating efficiency, that difference was not significant. For all panels, mating efficiency was measured after 20 h for solid media and 28 h for liquid media at 30°C. The results shown are representative of at least three independent experiments, and error bars represent the standard error of the mean (SEM).

Throughout the years, many aspects of ICE*Bs1* regulation and transmission have been characterized by using a domesticated strain of B. subtilis, incapable of forming a biofilm (10, 33). Here, we wanted to assess the real impact of biofilm on ICE*Bs1* transfer, and thus, without artificial induction. Mating assays were performed using NCIB3610, an undomesticated B. subtilis strain that forms strong and well-characterized biofilms and contains a kanamycin selection marker in ICE*Bs1*, as donor cells. Recipient cells were constructed by curing NCIB3610 of ICE*Bs1* (ICE*Bs1*^0^) as described previously (10). To discriminate between the effects of medium composition versus biofilm induction, pairs of complex rich media and defined minimal media were used as conjugative support. For each pair, one medium induces biofilm formation (LBGM and MSgg [see Materials and Methods]), while the other does not (LB and MSNc [see Materials and Methods]) (24, 25, 34) (see Fig. S1A in the supplemental material). Donor and recipient cells were mated in a 1:1 ratio, spotted, and incubated for 20 h on the various solid media. Conjugation efficiency was determined by plating serial dilutions of mating mixtures on selective media. Strikingly, we observed that the efficiency of exconjugant formation on biofilm-inducing media, compared to the conjugation on noninducing media, increased between 100-fold (minimal media; compare MSgg with MSNc) and 10,000-fold (rich media; compare LBGM with LB) (Fig. 1A). Importantly, these very high levels of transfer were obtained without the need to artificially activate ICE*Bs1* excision or add a DNA-damaging reagent such as mitomycin C. Similarly, formation of floating biofilms (pellicles) in liquid biofilm-inducing media induced high levels of ICE*Bs1* transfer, while media not inducing biofilms, in which cells are in planktonic form, showed no ICE*Bs1* transfer (Fig. 1A).

10.1128/mSphere.00473-18.1 FIG S1 LBGM and MSgg induce biofilm formation. WT (A) and *sinR* (B) donor and recipient cells were mated on LB (i), LBGM (ii), MSNc (iii), and MSgg (iv). Pictures were taken after incubation for 20 h at 30°C. Download FIG S1, PDF file, 0.3 MB. Copyright © 2018 Lécuyer et al. 2018 Lécuyer et al. This content is distributed under the terms of the Creative Commons Attribution 4.0 International license.

To confirm that increased conjugative ICE*Bs1* transfer explains the high level of exconjugant formation in biofilms, we performed mating assays on MSgg using *nicK* and *cwlT* deletion mutants: *nicK* and *cwlT* encode the relaxase and a cell wall hydrolase associated with ICE*Bs1* type IV secretion system, respectively (17, 35). As expected, transfer efficiencies dropped significantly for donors with either deletion (Fig. 1B), indicating that conjugation is the main HGT mechanism used for ICE*Bs1* acquisition in biofilm. Of note, on MSgg, *nicK* and *cwlT* mutants exhibited a residual frequency of exconjugant formation of 10^−5^: i.e., less than 1% of wild type (WT). Since extracellular DNA is a common feature of biofilm matrix, these exconjugants could result from acquisition of genetic material via transformation (27, 36). We tested this hypothesis by doing a conjugation assay with donor and recipient cells mutated for *comK*, the competence transcription factor of B. subtilis required for transformation (37), and *nicK* donor cells. As shown in Fig. S2 in the supplemental material, conjugation- and transformation-deficient cells did not produce any *kan^+^ cat*^+^ (exconjugants) cells on MSgg, confirming that natural transformations is responsible for approximately 1 out of 100 *kan^+^ cat*^+^ cells formed in biofilm. However, since the contribution of natural competency is negligible, mating assays performed with *comK* or WT cells showed similar efficiencies of exconjugant formation in biofilms (Fig. 1C).

10.1128/mSphere.00473-18.2 FIG S2 *nicK* residual exconjugant formation is due to natural transformation. Conjugation assays were performed with WT plus WT, *nicK* plus WT, and *nicK comK* plus *comK* cells on MSgg. Mating efficiency was measured after 20 h at 30°C. While the *nicK* plus WT assay showed a low level of exconjugant formation, the combination *nicK comK* plus *comK* led to complete abolition of transfer. Results shown are representative of at least three independent experiments, and error bars represent the SEM. Download FIG S2, PDF file, 0.1 MB. Copyright © 2018 Lécuyer et al. 2018 Lécuyer et al. This content is distributed under the terms of the Creative Commons Attribution 4.0 International license.

In B. subtilis, RapI and RecA are both capable of lifting inhibition on ICE*Bs1* excision and transfer (10). To evaluate which pathway regulates ICE*Bs1* transfer during biofilm formation, we performed mating assays on MSgg using *rapI* or *recA* null mutants as donors. We observed that neither led to a significant decrease of mating efficiency and that *recA* donor cells exhibited 5- to 10-fold higher mating efficiency. These results suggest either that a third unknown ICE*Bs1* activation pathway is active in biofilms or that both RapI and RecA pathways are redundant in biofilms. To discriminate between these two hypotheses, we tested the mating efficiency of a *rapI recA* donor strain and observed a 1,000-fold decrease in mating efficiency compared to the WT (Fig. 1D). These results suggest that both ICE*Bs1* activation pathways are active during biofilm formation and that they are redundant in biofilm. Interestingly, the frequencies of exconjugant formation with *nicK*, *cwlT*, or the *rapI recA* donors were comparable (Fig. 1B and D), further strengthening the notion that *rapI* and *recA* are the only ICE*Bs1* activators in biofilm.

### Biofilm allows for highly efficient conjugation in a low donor/recipient ratio.

**FIG 2:**
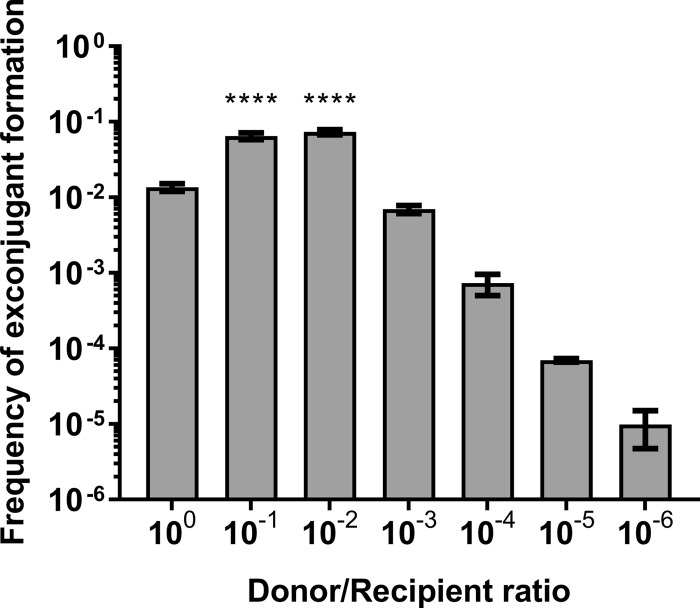
Lower donor/recipient ratio allows increased ICE*Bs1* transfer in biofilm. WT donor cells were diluted and mated with a fixed number of WT recipient cells on MSgg. Transfer efficiency was measured after 20 h at 30°C. Donor/recipient ratios of 1:10 and 1:100 show significantly more ICE*Bs1* transfer efficiency than the 1:1 ratio (one-way ANOVA; ****, *P* < 0.0001). The results shown are representative of at least three independent experiments, and error bars represent the SEM.

The 1:1 ratio of donor to recipient cells often used to assess conjugation *in vitro* is probably not frequently encountered in the environment. Therefore, we performed mating assays on MSgg using donor/recipient ratios ranging from 1:1 to 1:10^6^. We observed that exconjugant formation frequency was at its highest when recipient cells outnumbered donor cells by 10 to 100 times, whereas similar efficiencies were obtained between the 1:1 and the 1:10^3^ ratios and lower ratios showed decreased efficiency (Fig. 2). These observations can be partially explained by the ICE*Bs1*-encoded quorum-sensing system RapI-PhrI, since extracellular PhrI would not be sufficient to inhibit the RapI activator upon low donor cell density in the population. Alternatively, diffusion of PhrI could be hampered in the presence of the biofilm matrix. Of note, it is possible that newly formed exconjugants transfer ICE*Bs1* immediately after receiving it, which would compensate for the low initial level of donor cells. These results indicate that a small population of donor cells can efficiently transfer ICE*Bs1* in a biofilm community.

### Conjugation activation and biofilm formation are simultaneous.

**FIG 3:**
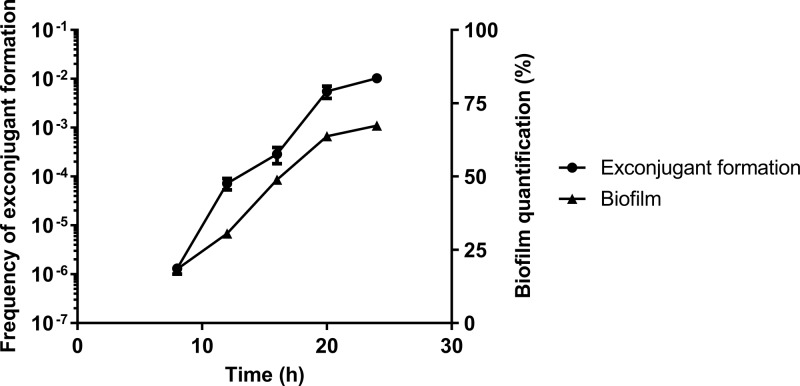
Biofilm formation and ICE*Bs1* conjugation activation are simultaneous. Recipient and donor cells harboring a P*_tapA_-yfp* fluorescent marker in the *amyE* locus were mated on MSgg. Biofilms were then harvested after 8, 12, 16, 20, and 24 h. For each time point, cells were used to quantify biofilm expression by fluorescence-activated cell sorter (FACS) and to assess mating efficiency. The results shown are representative of at least three independent experiments, and error bars represent the SEM.

Since biofilm formation positively influences ICE*Bs1* transfer, we examined the timing of conjugation in relation to biofilm formation. Mating efficiency was assessed on MSgg at different time points using donor and recipient cells carrying the P*_tapA_-yfp* reporter. In this reporter gene construction, the yellow fluorescent protein (YFP) is under the control of a matrix gene promoter, thus, allowing its expression concomitantly to matrix production, which can therefore be evaluated by quantification of fluorescent cells in a population by flow cytometry (24, 38). On solid MSgg medium, we observed a steady increase in both mating efficiency and biofilm matrix induction from 8 to 20 h after inoculation (Fig. 3), indicating a temporal correlation between biofilm formation and conjugation.

### Biofilm formation does not alter excision of ICE*Bs1*.

**FIG 4:**
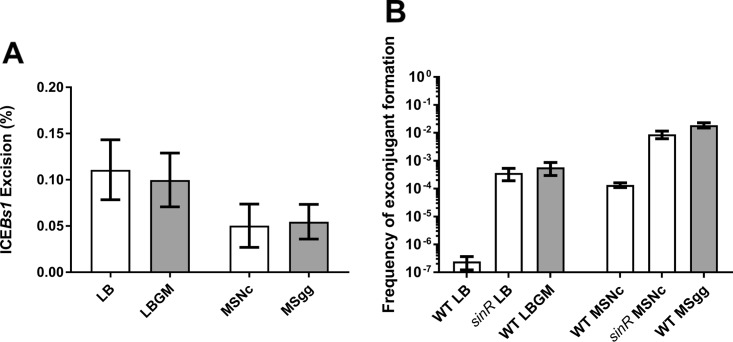
Matrix production is important for conjugation. (A) WT donor cells and ICE*Bs1*^0^ *attB*-down recipient cells were mated on LB, LBGM, MSNc, and MSgg for 20 h at 30°C, and the donor *attB* site was amplified by qPCR. There was no significant difference in the ICE*Bs1* excision rate when there was biofilm formation (Student’s *t* test). (B) *sinR* donor and recipient cells were mated on non-inducing media, and mating efficiency was compared to that of WT cells mated on non-biofilm-inducing (LB and MSNc [white bars]) and biofilm-inducing solid media (LBGM and MSgg [gray bars]). Mating efficiency was measured after 20 h at 30°C. *sinR* mutants led to higher ICE*Bs1* transfer efficiency compared to WT cells on noninducing media. For all panels, the results shown are representative of at least three independent experiments, and error bars represent the SEM.

The increase of conjugation throughout biofilm formation could be due to a gradual increase in ICE*Bs1* excision, which is under the control of several signaling pathways. To examine this hypothesis, we determined the ICE*Bs1* excision level under biofilm-forming conditions by monitoring the formation of the *attB* site in donor cells using quantitative PCR (qPCR). Since quorum sensing can influence ICE*Bs1* excision, we used a 1:1 donor/recipient ratio. Accordingly, we constructed recipient cells in which an erythromycin resistance cassette was inserted at the hybridization site of one of the qPCR primers. In this context, the recipient unoccupied *attB* site cannot be amplified, although the site remains functional (see Fig. S3 in the supplemental material). Surprisingly, there was no significant increase of ICE*Bs1* excision in donor cells on biofilm-inducing media compared to noninducing media (Fig. 4A). We also followed ICE*Bs1* excision in donor cells over time using the same time points as the conjugation assay previously described and observed low levels of excision between 4 h and 24 h (see Fig. S4 in the supplemental material). Of note, this method does not allow us to evaluate excision rates in exconjugants, which may lead to an underestimation of the subset of cells bearing excised ICE*Bs1*. However, these results show that biofilm formation does not alter ICE*Bs1* excision in donor cells.

10.1128/mSphere.00473-18.3 FIG S3 ICE*Bs1*^0^ *attB*-down recipient cells can acquire ICE*Bs1*, while the *attB* site cannot be amplified. (A) ICE*Bs1*^0^ *attB*-down (JSB18) cells were mated on MSgg with WT donor cells to assess their capacity to acquire ICE*Bs1*, despite an erythromycin cassette inserted near the *attB* site. Mating efficiency was measured after 20 h at 30°C. Statistical analysis showed no significant difference in mating efficiencies between the WT recipient cells and JSB18 cells (Student’s *t* test). Results shown are representative of at least three independent experiments, and error bars represent the SEM. (B) The *attB* site of a WT recipient cell (lane 1), JSB18 (lane 2), and a 1:1 mixture using JSB18 (lane 3) as a recipient was amplified by PCR using primers P333 and P358 (see Materials and Methods) and run on a 1% agarose gel. We were able to amplify the *attB* site from the WT recipient cells and the 1:1 mixture but not from JSB18 alone. Download FIG S3, PDF file, 0.1 MB. Copyright © 2018 Lécuyer et al. 2018 Lécuyer et al. This content is distributed under the terms of the Creative Commons Attribution 4.0 International license.

10.1128/mSphere.00473-18.4 FIG S4 Biofilm does not affect ICE*Bs1* excision over time. WT donor cells and ICE*Bs1*^0^ *attB*-down recipient cells were mated on MSgg, and the donor *attB* site was amplified by qPCR after 4, 8, 12, 16, and 20 h at 30°C. We did not detect an increase in ICE*Bs1* excision over time. Download FIG S4, PDF file, 0.1 MB. Copyright © 2018 Lécuyer et al. 2018 Lécuyer et al. This content is distributed under the terms of the Creative Commons Attribution 4.0 International license.

A second hypothesis to explain the positive effect of biofilm on conjugation is that the biophysical context provided by the extracellular matrix highly favors conjugative transfer. To test this idea, we used donor and recipient cells deleted for *sinR*, the transcriptional repressor of the *tapA-sipW-tasA* and *epsA–O* operons responsible for matrix production. A *sinR* mutant constitutively produces the biofilm matrix, even on non-biofilm-inducing media, with little effect on the upstream signaling pathways (27). This single mutation is sufficient to induce the formation robust biofilm, regardless of the media (Fig. S1B). As shown in Fig. 4B, transfer efficiency using *sinR* cells on both non-biofilm-inducing media was similar to that in WT cells on biofilm-inducing media. These results demonstrate that matrix production is sufficient to promote efficient transfer of ICE*Bs1* under non-biofilm conditions. To evaluate the importance of cell contact mediated by biofilm matrix for conjugative transfer versus the possible effects of *sinR* mutants or biofilm-inducing media on other cell processes, we examined ICE*Bs1* transfer in WT and *sinR* cells in planktonic (shaking) LB, LBGM, MSNc, and MSgg. Importantly, the WT in shaking biofilm-inducing media did not transfer ICE*Bs1* at all (LBGM) or transferred it at a lower rate than under biofilm conditions (floating pellicles) at similar donor and recipient cell densities (see Fig. S5 in the supplemental material). Also, *sinR* cells in all media and WT cells in MSgg rapidly clump despite agitation and show ICE*Bs1* transfer, suggesting that these cell aggregates mediated by matrix secretion are microenvironments favoring conjugative transfer (Fig. S5). Together, these results suggest that maximum transfer rates are obtained when biofilm matrix is produced and hold cells together.

10.1128/mSphere.00473-18.5 FIG S5 Conjugative transfer of ICE*Bs1* is inefficient under shaking conditions. WT, *eps tasA*, and *sinR* donor and recipient cells were mixed and inoculated in liquid LB, LBGM, MSNc, and MSgg. Tubes were then incubated for 20 h with shaking at 30°C, and exconjugant frequency was evaluated. The results shown are representative of at least three independent experiments, and error bars represent the SEM. Download FIG S5, PDF file, 0.1 MB. Copyright © 2018 Lécuyer et al. 2018 Lécuyer et al. This content is distributed under the terms of the Creative Commons Attribution 4.0 International license.

TasA was shown to bind cell together in the biofilm, and matrix exopolysaccharides were suggested to favor adhesion on neighboring cell chains in complex community development (39, 40). To strengthen the hypothesis that biofilm formation can provide a favorable context for conjugation by bringing cells closer or by stabilizing cell-cell contacts, we mated donor and recipient cells incapable of producing matrix (*epsA–O* and *tasA* mutants) on MSNc. We decided to emulate the binding effect provided by the extracellular matrix by adding 1% agarose, which is expected to move the matrix-deficient cells closer and stabilize their contact. We observed that addition of agarose to *eps tasA* mutants increased ICE*Bs1* transfer efficiency (see Fig. S6 in the supplemental material), suggesting that the polymer helps to stabilize the contact between cells the same way the extracellular matrix can, albeit to a lesser degree.

10.1128/mSphere.00473-18.6 FIG S6 Polymers can act as a surrogate biofilm in absence of extracellular matrix. WT cells and *epsA–O tasA* (*eps*) cells were resuspended in 1% agarose and mated on MSNc. Mating efficiency was measured after 20 h at 30°C. Statistical analysis showed a significant increase in ICE*Bs1* transfer efficiency when agarose was added to *eps tasA* cells, but not for the WT cells (Student’s *t* test; ****, *P* < 0.0001). Results shown are representative of at least three independent experiments, and error bars represent the SEM. Download FIG S6, PDF file, 0.1 MB. Copyright © 2018 Lécuyer et al. 2018 Lécuyer et al. This content is distributed under the terms of the Creative Commons Attribution 4.0 International license.

### Matrix production by recipient cells is important for optimum conjugation in biofilm.

**FIG 5:**
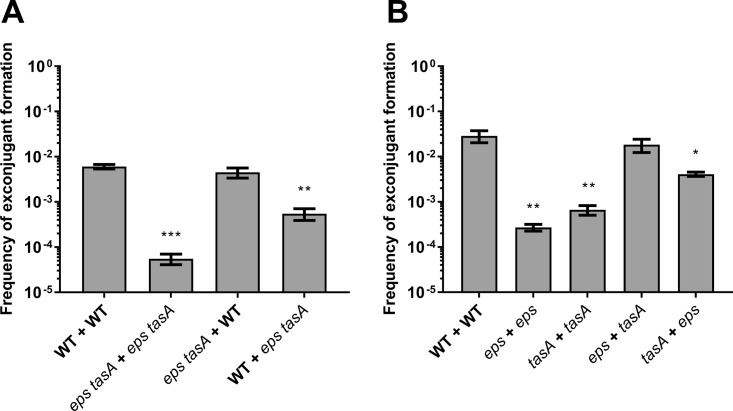
Both matrix components are important for ICE*Bs1* conjugation. (A) Donor and recipient cells deleted for the *epsA–O* (*eps*) and *tasA* operons were mated together or with WT cells on MSgg. The first genotype shown represents the donor genotype, while the second represent the recipient. Statistical analysis showed that absence of matrix and nonproduction from the recipient cells reduced significantly ICE*Bs1* transfer efficiency (one-way ANOVA; **, *P* < 0.01; ***, *P* < 0.001). (B) Donor and recipient cells mutated for either *eps* or *tasA* operon were mated on MSgg. Statistical analysis showed that absence of either exopolysaccharides or amyloid-like fibers in both donor and recipient decreases ICE*Bs1* transfer significantly. However, *eps* donors and *tasA* recipients can complement each other and restore the WT level of conjugation, while *tasA* donors and *eps* recipients are significantly different from WT pairs (one-way ANOVA; *, *P* < 0.05; **, *P* < 0.01). For both panels, mating efficiency was measured after 20 h at 30°C. The results shown are representative of at least three independent experiments, and error bars represent the SEM.

The results obtained with *sinR* mutants suggest that the biofilm matrix acts as a structure promoting cell-cell contacts and optimal ICE*Bs1* conjugative transfer. This hypothesis was further verified by carrying out mating assays with cells incapable of secreting matrix (i.e., *eps tasA* mutants). In B. subtilis, the *epsA–O* (*eps*) and *tasA* operons produce two major matrix components: exopolysaccharides and the TasA amyloid-like fibers, respectively (23). As shown in Fig. 5A, production of the extracellular matrix polymers was required for efficient ICE*Bs1* transfer on biofilm-inducing medium. Indeed, mating assays using mutant donor and recipient cells yielded transfer efficiencies similar to those observed on a minimal non-biofilm-inducing medium. Interestingly, while donor cells deficient for matrix production could still efficiently conjugate with WT recipient cells, mating WT donor with non-matrix-producing recipient cells significantly reduced transfer (Fig. 5A). This result suggests that the biofilm matrix production of the recipient cells is particularly important for efficient ICE*Bs1* transfer.

To examine the importance of both components for conjugation, mating assays with donor and recipient lacking either *eps* or *tasA* were performed. As shown in Fig. 5B, both components of the matrix were instrumental for efficient ICE*Bs1* transfer, stressing the importance of the matrix integrity for maximal conjugation. Various reports have shown that *eps* and *tasA* mutants can complement each other extracellularly to establish biofilm both *in vitro* and on plant roots (23, 24). Interestingly, combination of these mutants can also restore conjugation efficiency, but only when TasA is produced by donor cells (*eps* mutant) and the exopolysaccharides are produced by recipient cells (*tasA* mutant). The reverse combination resulted in a 5-fold reduction of transfer efficiency compared to WT cells (Fig. 5B). This result confirms that the extracellular matrix is essential for ICE*Bs1* transfer in biofilms and that matrix production by recipient cells is essential for optimal transfer.

## DISCUSSION

ICE*Bs1* regulation and its transfer mechanism have been thoroughly characterized in the last decade. However, as is also the case for most conjugative elements, its transfer between cells within a biofilm has not been previously studied. Here, we show that biofilm formation greatly increases conjugation of ICE*Bs1*, allowing for high-efficiency transfer in the absence of added DNA-damaging reagents.

Using donor cells carrying *nicK* and *cwlT* deletion mutations, both unable to transfer ICE*Bs1*, we validated that the high transfer observed in biofilm is due to conjugation events (Fig. 1B). However, we also observed a 10^−5^ background level of *kan^+^ cat^+^* “exconjugant” cell formation using donor cells with *nicK*, *cwlT*, or *rapI recA* deleted, the latter being unable to excise ICE*Bs1* (Fig. 1B and D). Further experiments allowed us to determine that these *kan^+^ cat^+^* cells arose by natural transformation, via transfer of the Kan^r^ gene present in ICE*Bs1* to recipient cells or the transfer of the *cat* gene present in recipient cells to donor cells (Fig. S2) (data not shown). These observations suggest that natural transformation contributes to approximately 1 out of 100 HGTs observed on MSgg and thus is also fairly efficient to promote gene transfer in biofilms formed by nondomesticated strains.

The minimal biofilm-inducing medium (MSgg) provided the highest frequency of ICE*Bs1* exconjugant formation (i.e., 10^−2^). Interestingly, *rapI* overexpression in a domesticated, non-biofilm-inducing B. subtilis strain also gives similar transfer efficiency, suggesting that it might be the upper limit for ICE*Bs1* transfer in a 1:1 ratio (10, 41). This transfer efficiency, obtained without artificial activation of ICE*Bs1* excision, demonstrates the high mobility of this element. ICE*Bs1* is therefore one of the ICEs transferring at the highest rate in the Firmicutes. Indeed, Staphylococcus aureus ICE*6013*, an ICE closely related to ICE*Bs1*, has a mating efficiency of around 10^−5^ (42). Tn*916*, an ICE found in a variety of Gram-positive bacteria, was reported to transfer at frequencies ranging from 10^−9^ to 10^−4^ (43, 44), while Streptococcus agalactiae TnGBS1 and TnGBS2 transfer at around 10^−5^ (45). Finally, Streptococcus thermophilus ICE*St3* transfers at a rate of 3.4 × 10^−6^, and only one conjugation event was ever reported for ICE*St1* (46). However, in the studies mentioned above, filter mating assays, which generally do not promote biofilm formation, were used to assess mating efficiencies. Our study demonstrates that to evaluate naturally relevant conjugation transfer of ICEs, the transfer rate within a biofilm must be examined.

Variable donor/recipient ratios ranging from 1:1 to 1:10^3^ in the mating population did not decrease the frequency of recipient cells acquiring ICE*Bs1*. This observation is very important, since it reflects how a genetic element, present in a small subset of an initial population, can propagate rapidly and efficiently. In fact, a ratio of 1:10 to 1:10^2^ led to a higher mating efficiency than a ratio of 1:1 (Fig. 2). This observation was previously reported for ICE*Bs1* (10), but was never explored in a biofilm-related setting. This increase in efficiency could be explained by the RapI-PhrI quorum-sensing system, since a smaller amount of donor cells in the biofilm leads to a low level of PhrI in the extracellular environment, thus favoring the action of the RapI activator (10). A similar system was described in Enterococcus faecalis, where a lower donor cell density led to higher transfer efficiency of conjugative plasmids pAD1 and pAM373, which both encode a secreted conjugation inhibitor (iAD1 and iAM373, respectively) (47). However, quorum-sensing regulation of excision has been observed only in a limited subset of genetic elements. For many others, DNA damage and/or environmental conditions such as reaching stationary phase or the presence of subinhibitory concentrations of antibiotics trigger ICE excision (48–50). ICE*Bs1* excision is also induced by DNA damage via the *recA* pathway, but *recA* donors showed better conjugation efficiency than WT donors (Fig. 1D). A similar increase of mating efficiency with a *recA* donor was observed previously (51). This result can be explained by the poor growth of *recA* mutants, known to have a slower doubling time (52). While cells were initially mated at a 1:1 donor/recipient ratio, we observed that after 20 h, the ratio had become approximately 1:50 (see Table S1 in the supplemental material), leading to a higher mating efficiency (Fig. 2).

10.1128/mSphere.00473-18.7 TABLE S1 *recA* and WT donor cell counts after 20 h. Download Table S1, PDF file, 0.1 MB. Copyright © 2018 Lécuyer et al. 2018 Lécuyer et al. This content is distributed under the terms of the Creative Commons Attribution 4.0 International license.

Somewhat surprisingly, we observed that biofilm formation does not induce excision of ICE*Bs1* in donor cells. Despite the low excision rate (∼0.2%), transfer levels in biofilms were similar to those obtained with donor cells overexpressing *rapI*, for which excision rates reach approximately 90% (10, 51). Of note, ∼0.2% is more than 10 times as high as the excision rate observed in an uninduced domesticated strain (51). ICE*Bs1* is known to replicate in a rolling circle and can be present in multiple copies in the donor cell (16). Thus, we hypothesize that under biofilm-forming conditions, ICE*Bs1* rapidly reintegrates into its host chromosome following replication. The extrachromosomal copies would then be transferred to recipient cells, explaining the efficient transfer despite low excision levels. Another hypothesis underlying the high conjugative transfer of ICE*Bs1* in biofilm could be the presence of abundant cell chains. Indeed, ICE*Bs1* transfers exceptionally well through bacterial chains (53), and these structures are frequently found in biofilms, which could help propagate ICE*Bs1* much more efficiently (23, 34). Considering the very low excision rate and the high conjugative transfer, it is also extremely likely that a single donor cell can propagate ICE*Bs1* to multiple recipients in biofilms. It is also likely that once a recipient receives ICE*Bs1*, it can immediately become a donor, further spreading it in the population. Importantly, the excision rate is not necessarily correlated with conjugation efficiency, as shown for Tn*916* (43).

Many conjugative elements encode surface factors that stabilize the contact between donor and recipient cells, such as conjugative pili in Gram-negative bacteria and adhesins in Gram-positive bacteria (54, 55). While it is unknown whether ICE*Bs1* encodes surface factors, its low transfer efficiency in liquid compared to solid media suggests that no such factors are expressed under these conditions (56). Here, we have shown that both components of the extracellular matrix are required for the positive effect of biofilm on conjugation, suggesting that these polymers could help stabilize the donor-recipient pair and compensate for the lack of adhesion factors of ICE*Bs1*. Other conjugative elements that are not known to encode adhesion factors, such as pCW3 and Tn*916*, are found in the biofilm-forming bacteria Clostridium perfringens and E. faecalis, respectively (57–59). Lack of adhesion factors in those elements could be compensated for by the ability of their host cells to form biofilms.

Experiments with single and double biofilm mutants allowed us to determine the individual importance of both matrix components in conjugation. Interestingly, matrix production from the recipient cells, but not from the donor cells, is likely essential for efficient transfer (Fig. 5A). This observation could be explained by the fact that recipients that do not produce matrix will not form cell chains, and thus, lead to less-efficient ICE*Bs1* transfer. It also suggests that cells within a biofilm might be able to receive ICEs from either biofilm- or non-biofilm-forming cells, making the biofilm a very receptive environment for genetic element transfer. These results allow us to better understand conjugative element dynamics in natural and clinical environments, where biofilms are ubiquitous. Biofilm matrix could thus have a considerable impact on the dissemination of mobile genetic elements, such as for the clinically important bacteria S. aureus and C. difficile, which can acquire multiple-antibiotic resistance through ICEs (60, 61).

## MATERIALS AND METHODS

### Strains and media.

The strains used in this study are derivatives of the ancestor strain NCIB3610 (see Table S2 in the supplemental material). The different media used for mating assays are LB (Luria Bertani; 1% tryptone, 0.5% yeast extract, 0.5% NaCl), LBGM (LB plus 1% glycerol and 0.1 mM MnCl_2_ [25]), MSNc (5 mM potassium phosphate buffer, pH 7, 0.1 M morpholinepropanesulfonic acid [MOPS], pH 7, 2 mM MgCl_2_, 0.05 mM MnCl_2_, 1 µM ZnCl_2_, 2 µM thiamine, 700 µM CaCl_2_, 0.2% NH_4_Cl, 0.5% cellobiose) (24), and MSgg (5 mM potassium phosphate buffer, pH 7, 0.1 M MOPS, pH 7, 0.025 mM FeCl_3_, 2 mM MgCl_2_, 0.05 mM MnCl_2_, 1 µM ZnCl_2_, 2 µM thiamine, 700 µM CaCl_2_, 0.5% glycerol, 0.5% glutamate) solidified with 1.5% agar (34). Media did not affect significantly bacterial growth, with biofilm-inducing media leading to slightly more yield compared to noninducing media (see Table S3 in the supplemental material). When needed, the following antibiotics were added to media: MLS (1 μg ml^−1^ erythromycin, 25 μg ml^−1^ lincomycin), spectinomycin (100 μg ml^−1^), tetracycline (10 μg ml^−1^), chloramphenicol (5 μg ml^−1^), and kanamycin (10 μg ml^−1^).

10.1128/mSphere.00473-18.8 TABLE S2 Bacillus subtilis strains used. Download Table S2, PDF file, 0.2 MB. Copyright © 2018 Lécuyer et al. 2018 Lécuyer et al. This content is distributed under the terms of the Creative Commons Attribution 4.0 International license.

10.1128/mSphere.00473-18.9 TABLE S3 Donor and recipient cell counts in different media after 20 h. Download Table S3, PDF file, 0.1 MB. Copyright © 2018 Lécuyer et al. 2018 Lécuyer et al. This content is distributed under the terms of the Creative Commons Attribution 4.0 International license.

### Strain construction.

Most strains were made by transferring genetic constructs present in domesticated strains in NCIB3610, using SPP1-mediated generalized transduction (62). JMA348 (ICE*Bs1*::*kan*), CAL51 [(*rapI phrI*)*342*::*kan*], JMA208 (*immR*::*cat*), and CAL419 (ICE*Bs1*^0^ *comK*::*cat*) were kind gifts from Alan D. Grossman (Massachusetts Institute of Technology, MA), and 3610 ICE*Bs1*^0^ strains were cured from ICE*Bs1* and verified as described in reference 10. Briefly, MG9 (3610 *immR*::*cat*) was inoculated in LB, grown for 4 h, diluted at an optical density at 600 nm (OD_600_) of 0.01 in fresh LB, and grown overnight at 37°C. The culture was then diluted back to an OD_600_ of 0.01 in fresh LB and grown until the culture reached an OD_600_ of 1. The cells were then plated on LB agar and grown overnight at 37°C, and colonies were streaked on LB with or without chloramphenicol. Colonies that lost the resistance were then PCR verified for ICE*Bs1* excision with the following primers (5′→3′): P197 (GAC GAA TAT GGC AAG CCT ATG TTA C) and P198 (GGG TAT ACA ATC ATG GGT GAT CGA G).

Long-flanking homology PCR was used to insert a spectinomycin cassette between *ycbU* and *lmrB* and to create the recipient used for qPCR (JSB18). The following primers (5′→3′) were used for the *ycbU-lmrB*::*spec* insertion: P246 (CCA TTG ATG TGA AGG AAT GGG GCG TA), P247 (CGT TAC GTT ATT AGC GAG CCA GTC ATG TTT ACT TGT GGA TCG TTT TCG CCG), P248 (CAA TAA ACC CTT GCC CTC GCT ACG CCT GAA CAC TAG TCA GGG GCT TTT CA), and P249 (GGC TTA GTC CTC ACT GCA TTT GCA TC). The following primers (5′→3′) were used for the *attB*-down::*erm* deletion: P328 (CCG TTG GTC AAG CGG TTA AG), P329 (GAG GGT TGC CAG AGT TAA AGG ATC TAT TAT TGA GAT GCG GCC GAG), P330 (CGA TTA TGT CTT TTG CGC AGT CGG CGT GTG GAA AAT ACG GCT ATG GG), and P331 (AGT AAG CTT ATT CCA CCC ACT G). PCR products were then introduced in B. subtilis 168 by natural competency (63), verified by PCR, and transferred in derivatives of B. subtilis NCIB3610 by SPP1-mediated generalized transduction (62).

### Mating assays.

Donor and recipient cells were grown in 3 ml LB broth at 37°C overnight, diluted at an OD_600_ of 1.5 in LB, and mixed at a 1:1 ratio (or the specified ratio [Fig. 2]). The mixture was then centrifuged for 3 min at 5,000 rpm. The cell pellet was resuspended in 50 µl LB, and 10 µl was dropped onto the appropriate medium and incubated for 20 h (or the time specified [Fig. 3]) at 30°C, which is the temperature at which B. subtilis biofilm grows efficiently. For mating assays in liquid, 10 µl of bacterial mixture was used to inoculate 3 ml of medium for shaking conditions, while 3.33 µl was used to inoculate 1 ml of medium found in a 24-well plate for pellicle mating; both were also done at 30°C. Pellicles were incubated for 28 rather than 20 h, since the growth dynamic is slower under nonshaking conditions. For mating assays using agarose, cells were resuspended in 50 µl of warm (55°C) 1% molecular-grade agarose before being dropped on agar medium. Cells were then collected with 1 ml LB broth and sonicated at 30% amplitude for 20 s two times for cells grown for 20 h on biofilm-inducing media. Microscopy observation allowed us to determine that sonication was sufficient to obtain single cells. Cells were then serially diluted and plated on LB with the appropriate antibiotics. Donor, recipient, and exconjugant CFU were then counted. We expressed the frequency of exconjugant formation as a function of the number of recipient CFU (number of exconjugant CFU divided by the number of recipient CFU), because the starting donor amount varied in some experiments (Fig. 2).

### Flow cytometry.

Mating assays were performed on MSgg as described above, using the FL60 and FL63 strains. For each time point, three biofilms were harvested with 500 µl phosphate-buffered saline (PBS: 137 mM NaCl, 2.7 mM KCl, 8 mM Na_2_HPO_4_, 2 mM KH_2_PO_4_, pH 7.4) and disrupted with up and down pipetting. Subsequent steps were performed as described previously (38). Flow cytometry analysis was performed on a BD FACSJazz (BD Biosciences).

### ICE*Bs1* excision quantification.

ICE*Bs1* excision was evaluated using qPCR. Donor and recipient cells were mated on MSgg as described above, and cells were harvested and flash-frozen at the appropriate time point. Genomic DNA was extracted using the BioBasic genomic DNA extraction kit, and qPCR was performed on the *attB* site (created when ICE*Bs1* is excised from the chromosome) using the following primers (5′→3′): P358 (GCC TAC TAA ACC AGC ACA AC) and P333 (AAA GGT GGT TAA ACC CTT GG). Since the recipient strain (JSB18) contains an erythromycin resistance cassette in the hybridization site of the P333 primer, amplification is only possible for donor cells in which ICE*Bs1* is excised. qPCR on a chromosomal chloramphenicol resistance cassette present only in the donor genome was performed for normalization (threshold cycle [Δ*C_T_*]) of donor cells, using the following primers (5′→3′): P363 (AGA ACT GGT TAC AAT AGC GAC GGA GAG) and P366 (CCC CGA ACC ATT ATA TTT CTC TAC ATC AGA AAG G). The percentage of excision is calculated as the ΔΔ*C_T_* using the culture of the ICE*Bs1*^0^ *ylnF*/*yboA*::Tn*917*::*amyE*::*cat* control strain grown under the same conditions, which is considered as being 100% excised.

### Stereomicroscopy.

Donor and recipient WT and *sinR* cells were mated on LB, LBGM, MSNc, and MSgg as described above. Photographs of colonies were taken after 20 h at 30°C with a Leika M165 FC (Leika).

### Statistical analyses.

Statistical analyses were performed using GraphPad Prism 7. Comparisons were done using Student's *t* test or one-way analysis of variance (ANOVA) followed by Tukey’s multiple-comparison test, both with 95% confidence intervals.
