# Supplementary figures and images for: Biofilm Formation Drives Transfer of the Conjugative Element ICEBs1 in Bacillus subtilis
Source: mSphere. 2018 Sep 26;3(5):e00473-18. doi: 10.1128/mSphere.00473-18 (PMC6158512; doi:10.1128/mSphere.00473-18)

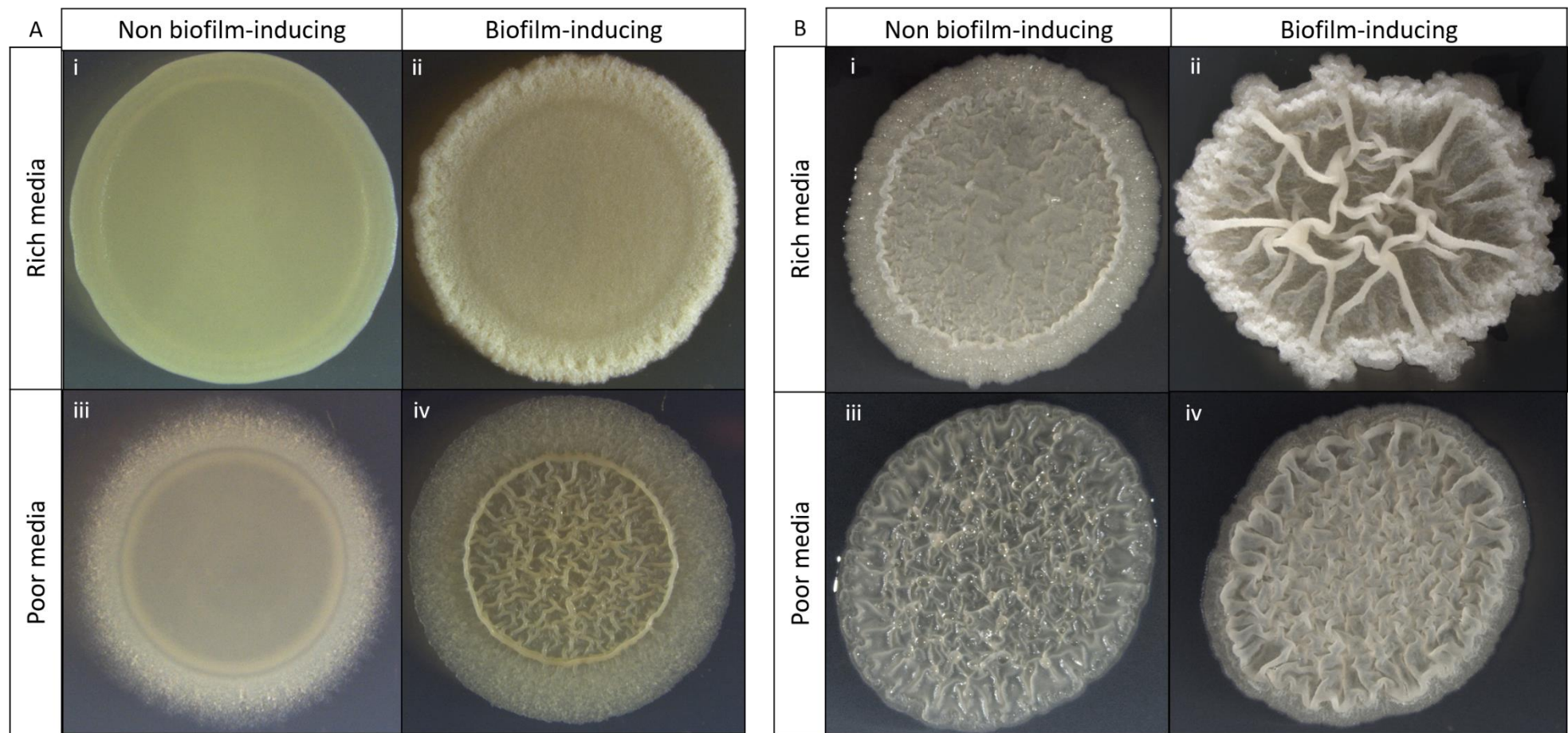

**Fig. S1**

Supplement: FIG S1 [file sph005182649sf1.pdf]

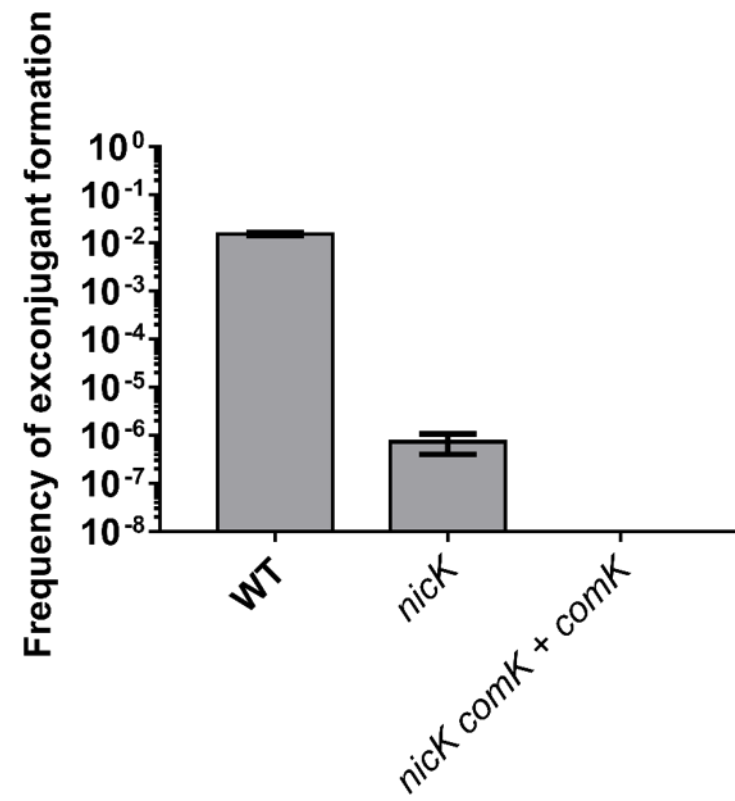

Fig. S2

Supplement: FIG S2 [file sph005182649sf2.pdf]

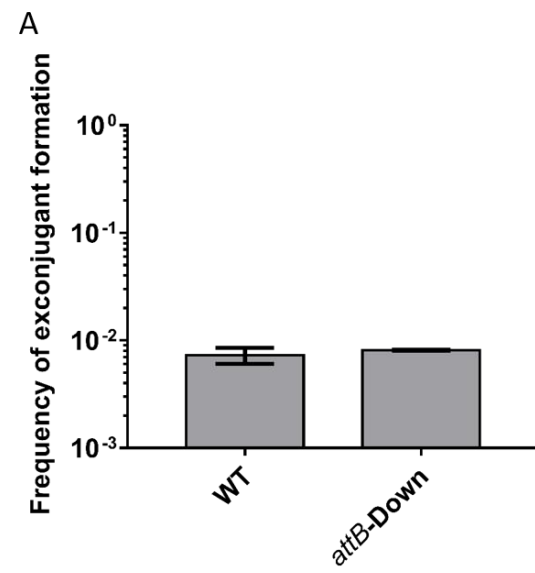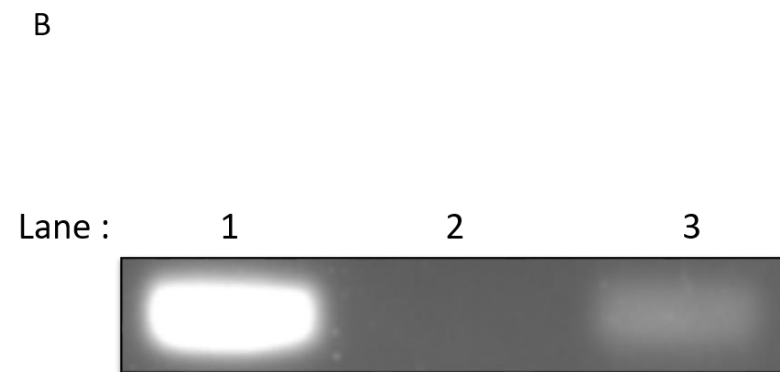

Fig. S3

Supplement: FIG S3 [file sph005182649sf3.pdf]

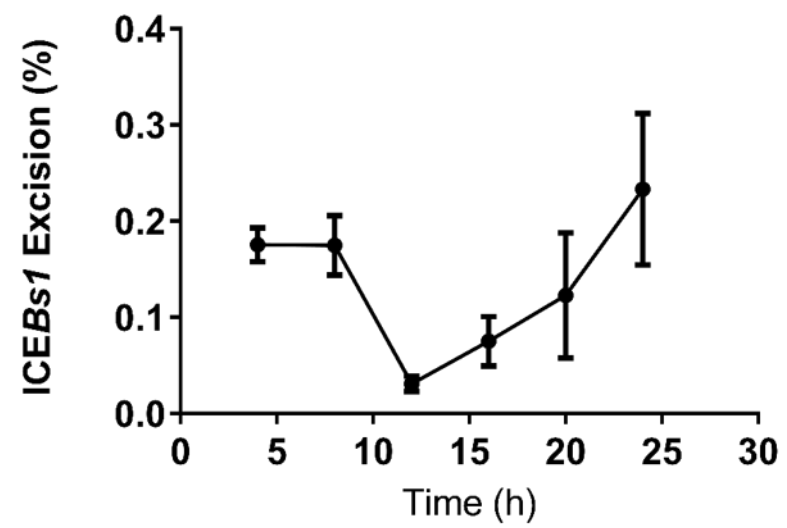

**Fig. S4**

Supplement: FIG S4 [file sph005182649sf4.pdf]

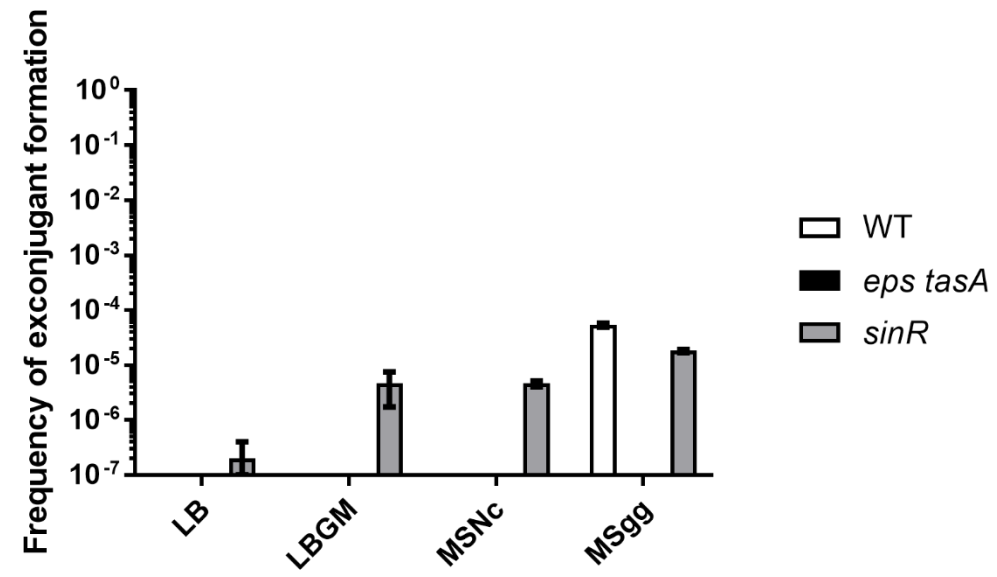

**Fig. S5**

Supplement: FIG S5 [file sph005182649sf5.pdf]

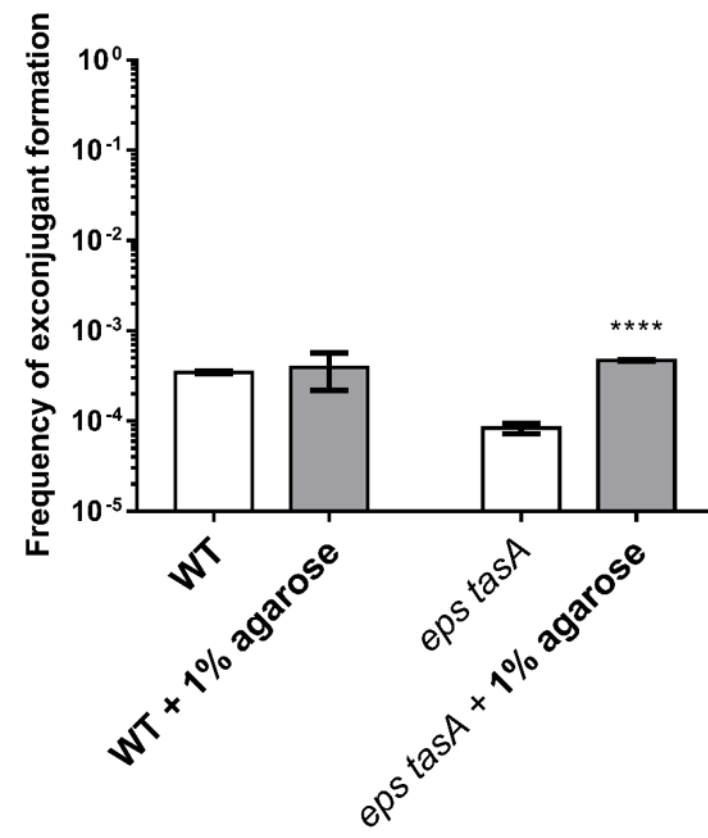

**Fig. S6**

Supplement: FIG S6 [file sph005182649sf6.pdf]
