# Supplementary material for: Biofilm Formation Drives Transfer of the Conjugative Element ICEBs1 in Bacillus subtilis
Source: mSphere. 2018 Sep 26;3(5):e00473-18. doi: 10.1128/mSphere.00473-18 (PMC6158512; doi:10.1128/mSphere.00473-18)
Supplement: TABLE S1 [file sph005182649st1.pdf]

Table S1

	Concentration at 20 h (CFU/ml)
WT donors (FL62)	$6.93 \times 10^{8} \pm 2.71 \times 10^{8}$
WT recipient (MG25)	$1.03 \times 10^{9} \pm 3.56 \times 10^{8}$
recA donors (FL92)	$2.30 \times 10^{7} \pm 2.00 \times 10^{6}$
WT recipient (MG25)	$1.33 \times 10^{9} \pm 4.44 \times 10^{7}$
