# Supplementary material for: Biofilm Formation Drives Transfer of the Conjugative Element ICEBs1 in Bacillus subtilis
Source: mSphere. 2018 Sep 26;3(5):e00473-18. doi: 10.1128/mSphere.00473-18 (PMC6158512; doi:10.1128/mSphere.00473-18)
Supplement: TABLE S2 [file sph005182649st2.pdf]

Strain	Genotype	Construction
168	**Domesticated strain**	Lab stock
3610	**Wild type** Undomesticated strain	Lab stock
VCL28	168 *ycbU-lmrB*::*spec*	See Methods
MG28	ICE*Bs1-kan*	JMA384[1] in 3610
FL62	ICE*Bs1-kan ycbU-lmrB*::*spec*	VCL28 in MG28
MG13	ICE*Bs1*^{0}	JMA208[1] in 3610 and curing
MG25	ICE*Bs1*^{0} *ylnF*/*yboA*::Tn*917*::*amyE*::*cat*	PB514[#] in MG13
FL66	ICE*Bs1*^{0} *comK*::*cat*	CAL419[1] in MG13
FL80	ICE*Bs1-kan ycbU-lmrB*::*spec nicK*::*erm*	BKE04870[2] in FL62
FL83	ICE*Bs1-kan ycbU-lmrB*::*spec cwlT*::*erm*	BKE04970[2] in FL62
FL104	ICE*Bs1*^{0} *comK*::*cat ycbU-lmrB*::*spec*	VCL28 in FL66
FL103	ICE*Bs1-kan nicK*::*erm*	BKE04870[2] in MG28
FL105	ICE*Bs1-kan nicK*::*erm comK*::*cat*	CAL419[1] in FL103
FL106	ICE*Bs1-kan comK*::*cat*	CAL419[1] in MG28
FL91	(*rapI phrI*)342::*kan ycbU-lmrB*::*spec*	CAL51[1] and VCL28 in 3610
FL92	ICE*Bs1-kan ycbU-lmrB*::*spec recA*::*erm*	BKE16940[2] in FL62
FL96	(*rapI phrI*)342::*kan ycbU-lmrB*::*spec recA*::*erm*	BKE16940[2] in FL91
FL63	ICE*Bs1-kan amyE*::P*tapA-yfp*	CA018[3] in MG28
FL60	ICE*Bs1*^{0} *ylnF*/*yboA*::*Tn917*::*amyE*::*cat lacA*::P*tapA-yfp*	DL821[4] in MG25
JSB7	168 *attB*-down::*erm*	See Methods
JSB18	ICE*Bs1*^{0} *attB*-down::*erm*	This study
JSB19	ICE*Bs1-kan ylnf*/*yloA*::*Tn917*::*amyE*::*cat*	PB514[#] in MG28
FL93	ICE*Bs1-kan sinR*::*spec*	PB194[#] in MG28
FL94	ICE*Bs1*^{0} *ylnF*/*yboA*::Tn*917*::*amyE*::*cat sinR*::*spec*	PB194[#] in MG25
MG44	ICE*Bs1-kan tasA*::*erm epsA-O*::*tet*	SSB569[#] in MG28
FL77	ICE*Bs1-kan ycbU-lmrB*::*spec tasA*::*erm eps*::*tet*	VCL28 in MG44
MG43	ICE*Bs1*^{0} *ylnF*/*yboA*::Tn*917*::*amyE*::*cat tasA*::*erm epsA-O*::*tet*	SSB569[#] in MG38

FL87	ICE*Bs1-kan ycbU-lmrB*::*spec epsA-O*::*tet*	SSB569# in FL62
FL90	ICE*Bs1*^{0} *ylnF*/*yboA*::Tn*917*::*amyE*::*cat epsA-O*::*tet*	SSB569# in MG25
FL88	ICE*Bs1-kan ycbU-lmrB*::*spec tasA*::*erm*	SSB569# in FL62
MG38	ICE*Bs1*^{0} *ylnF*/*yboA*::Tn*917*::*amyE*::*cat tasA*::*erm*	SSB569# in MG25

*Unless indicated, all strains are derivatives of *B. subtilis* NCIB 3610. Antibiotics: spectinomycin (spec), chloramphenicol (cat), erythromycin/lincomycin (erm), tetracycline (tet), kanamycin (kan).

#These strains come from the Kolter and/or Beauregard lab stock.

1- Auchtung, J. M., Lee, C. A., Monson, R. E., Lehman, A. P., Grossman, A. D. (2005) Regulation of a *B. subtilis* mobile genetic element by intercellular signaling and the global DNA damage response. *Proc. Natl. Acad. Sci. USA* **102**, 12554–12559.

2- Koo, B. M. *et al.* (2017) Construction and analysis of two genome-scale deletion libraries for *Bacillus subtilis*. *Cell Syst.* **4**, 291-305.e7.

3- Vlamakis, H., Aguilar, C., Losick, R., Kolter, R. (2008) Control of cell fate by the formation of an architecturally complex bacterial community. *Genes & Dev.* **22**, 945-953.

4- López, D., Vlamakis, H., Losick, R., Kolter, R. (2009) Cannibalism Enhances Biofilm Development in *Bacillus subtilis*. *Mol. Microbiol.* **74**, 609-618.
