# Supplementary material for: Biofilm Formation Drives Transfer of the Conjugative Element ICEBs1 in Bacillus subtilis
Source: mSphere. 2018 Sep 26;3(5):e00473-18. doi: 10.1128/mSphere.00473-18 (PMC6158512; doi:10.1128/mSphere.00473-18)
Supplement: TABLE S3 [file sph005182649st3.pdf]

Table S3

	LB	LBGM	MSNc	MSgg
3610 ICE*Bs1-kan ycbU-lmrB*::*spec* (Donor)	$3.97x10^8$ ± $4.89x10^7$	$1.00x10^9$ ± $2.62x10^8$	$3.60x10^8$ ± $5.33x10^7$	$9.27x10^8$ ± $1.64x10^8$
3610 ICE*Bs1*^{0} *ylnF*/*yboA*::Tn917::*amyE*::*cat* (Recipient)	$7.70x10^8$ ± $2.20x10^8$	$1.50x10^9$ ± $4.00x10^8$	$4.87x10^8$ ± $3.11x10^7$	$1.37x10^9$ ± $1.78x10^8$

Concentration is expressed in CFU/ml
